# The Effect of Recombinant Human Iduronate-2-Sulfatase (Idursulfase) on Growth in Young Patients with Mucopolysaccharidosis Type II

**DOI:** 10.1371/journal.pone.0085074

**Published:** 2014-01-13

**Authors:** Zbigniew Żuber, Agnieszka Różdżyńska-Świątkowska, Agnieszka Jurecka, Anna Tylki-Szymańska

**Affiliations:** 1 Department of Pediatrics, S. Louis Regional Children’s Hospital, Cracow, Poland; 2 Anthropology Laboratory, The Children’s Memorial Health Institute, Warsaw, Poland; 3 Department of Molecular Biology, University of Gdańsk, Gdańsk, Poland; 4 Department of Medical Genetics, The Children’s Memorial Health Institute, Warsaw, Poland; 5 Department of Pediatrics, Nutrition and Metabolic Diseases, The Children’s Memorial Health Institute, Warsaw, Poland; Baylor Research Institute, United States of America

## Abstract

Mucopolysaccharidosis type II (MPS II; Hunter syndrome) is an X-linked, recessive, lysosomal storage disorder caused by deficiency of iduronate-2-sulfatase. Early bone involvement leads to decreased growth velocity and short stature in nearly all patients. Our analysis aimed to investigate the effects of enzyme replacement therapy (ERT) with idursulfase (Elaprase) on growth in young patients with mucopolysaccharidosis type II. Analysis of longitudinal anthropometric data of MPS II patients (group 1, n = 13) who started ERT before 6 years of age (range from 3 months to 6 years, mean 3.6 years, median 4 years) was performed and then compared with retrospective analysis of data for MPS II patients naïve to ERT (group 2, n = 50). Patients in group 1 received intravenous idursulfase at a standard dose of 0.58 mg/kg weekly for 52–288 weeks. The course of average growth curve for group 1 was very similar to growth pattern in group 2. The average value of body height in subsequent years in group 1 was a little greater than in group 2, however, the difference was not statistically significant. In studied patients with MPS II, idursulfase did not appear to alter the growth patterns.

## Introduction

Mucopolysaccharidosis type II (MPS II, Hunter syndrome, OMIM# 309900) is caused by the deficiency of the enzyme iduronate-2-sulfatase (I2S; EC 3.1.6.13) that is responsible for breaking down heparan and dermatan sulfate (HS and DS) within the cells [1]. It is a rare and life-limiting X-linked recessive disorder that affects approximately 1 in 77,000 newborn boys [2], [3]. Girls are affected rarely [4].

MPS II leads to profound disruption in the normal mechanism of growth and development and a major feature of this disorder is abnormal bone and cartilage development [1]. These abnormalities arise from a lack of skeletal remodeling, disordered endochondral and intramembranous ossification, disruption of normal elastogenesis and the infiltration by GAGs [5]. It has been shown that inflammation secondary to GAG accumulation is a critical aspect of MPS disorder and contributes to the bone disease [6].

Infants with MPS II appear normal at birth, and early developmental milestones may also be within normal range; in the first years of life height of most patients with MPS II is above 50^th^ percentile and in some cases is over the 97^th^ percentile. However, growth velocity decreases with age: by the age of 8 years, height is below the 3^rd^ percentile and nearly all patients exhibit growth retardation before puberty [7], [8].

Currently, both hematopoietic stem cell transplantation (HSCT) and enzyme replacement therapy (ERT) using idursulfase (recombinant human 2IS, Elaprase, Shire Human Genetic Therapies, Inc., Lexington, MA, USA) are available for MPS II. ERT has been shown to be effective in ameliorating some of the clinical manifestation of MPS disease. Long-term effect of ERT on the natural history of growth patterns is less clear and detailed data on the impact of ERT on growth are very limited [9]–[11].

The aim of this study was to analyze the effect of ERT with idursulfase on growth in 13 young MPS II patients who began ERT before the age of 6 years.

## Materials and Methods

### Study design

The study objective was to evaluate the effectiveness of idursulfase on body height in young patients with MPS II by comparing a natural growth pattern in children with MPS II naïve to ERT with growth of MPS II patients who started ERT before 6 years of age.

All patients were enrolled at The Children’s Memorial Health Institute (CMHI) in Poland. The clinical decisions for the patients were made by the clinicians/researchers (ATS, ZZ).

### Patients

All patients enrolled in the study had to have a diagnosis of MPS II confirmed by the biochemical determination of iduronate-2-sulfatase deficiency in leukocytes and by molecular analysis. The patients were divided in two following groups:

**Group 1.** The first group consisted of 13 patients who had a diagnosis of MPS type II confirmed by biochemical and molecular analysis and started ERT before the age of 6 years. The demographic characteristics of these thirteen MPS II patients are listed in Table 1.

**Table 1:** Patient characteristics (demographic, molecular characteristics and clinical phenotypes).

Patient	Age (y.)	Age (y.)	Age (y.)	Total	Mutations	Phenotype
No.	Diagnosis	Baseline	Current	idursulfase		
				exposure (week)		
1	2	5.75	11	198	deletion of IDS gene	neurological
2	4	5.5	11	159	c.1001 A>C	neurological
3	4	4.5	7.5	156	intragenic inversion	neurological
4	3	4	8	164	c.998C>T	neurological
					p.Ser333Leu	
5	2	4	8	188	c.950_95delCT	neurological
6	3	3.5	8	188	c.998C>T	neurological
7	3	3	7	188	intragenic inversion	neurological
8	3	4	8	188	c.1135_1136insGTAA	neurological
					p.Pro379Argfsx8	
9	3	4	8	164	Ivs2+29G→C	neurological
10	1	2	deceased	52	deletion xq28	neurological
11	2	3	5	92	c.1007G>A	neurological
					p.Gly336Glu	
12	1/12	3/12	6	288	c.1568A>G	attenuated
13	3	3	6.5	164	c.998C>T	neurological
					p.Ser333Leu	

**Group 2.** The second group consisted of 50 patients who were naïve to ERT.

Since 1989 till 2009, a longitudinal growth study was performed at The Children’s Memorial Health Institute (CMHI), Warsaw, Poland. The study population contained patients with MPS II (n = 50) aged from 0.5 to 21 years (mean 8.2 years, median 8.5 years). All patients had the severe (neurological) form of disease. All patients were born at term, and presented with the typical clinical features of MPS and had a diagnosis of MPS type II confirmed by biochemical and molecular analysis (median age of diagnosis 3 years).

All patients were naïve to ERT during the time of the study. These patients can be divided into two groups: 1) patients who have never been treated with idursulfase as they died before the introduction of ERT in Poland and 2) patients who received ERT later in life, but had anthropometric measurements performed before the introduction of ERT. In general, patients in group 1 were more recently diagnosed than patients in group 2, but there was no selection bias.

### Ethical consideration

The protocol was approved by the human-subjects institutional review board at The Children’s Memorial Health Institute. Written informed consent had to be provided by the parents or legal guardians.

### Elaprase treatment

All patients received weekly intravenous infusions idursulfase (Elaprase, Shire Human Genetic Therapies, Lexington, MA) at a standard dose of 0.58 mg/kg. The treatment was done according to the standard care and was administered by the clinicians/researchers (ATS, ZZ).

### Evaluation of efficacy

The primary efficacy endpoint variable was body height. This efficacy measure was taken once periodically during treatment (at weeks 24, 48, 72, 96 weeks and annually after the 96^th^ week). Until the age of 3 years length was measured in the supine position using a liberometer (accuracy to 1 mm). The same measurements of the older children were performed as standing height using a stadiometer (accuracy to 1 mm). Weight was measured using an electronic scale accurate within 0.05 kg. A non-stretchable tape was used to assess head and chest circumference (accuracy to 5 mm). The same anthropologist performed all assessments.

### Statistical analysis

The data for group 1 and 2 was divided into 24 calendar age classes. Children between 6 and 36 months of life were then categorized into age groups of 3 months, while patients between 3 and 21 years of life into groups of 1 year. The degree and direction of deviations of studied features in children with MPS II were analyzed using the data standardization method and the calculated values were presented as z-scores.

All measurements were standardized for age and gender using the Polish body growth reference charts [12]. The resulting z-scores were used in all calculations. Descriptive statistics, including means and z-scores were calculated using Statistica 7 PL (Statsoft, Poland). Two-tailed t-tests were used to compare mean standardized values for body height between groups 1 and 2.

The study has been reported according to STROBE guidelines.

## Results

### Patients

Thirteen patients (patients 1–13, Table 1) were born at term and received a diagnosis of MPS II at a mean age of 2.5 years (ranging from 1 month to 4 years, median 3 years). They began ERT with idursulfase at a mean age of 3.6 years (range from 3 months to 6 years, median 4 years). All were Caucasian. Twelve patients were classified as neurological (92.3%) and one as attenuated (7.7%).

Of the 13 enrolled patients, all completed the study. All patients received weekly intravenous infusions of idursulfase for 52 to 288 weeks. All 13 patients completed at least 52 weeks of treatment, 1 patient (7.7%) completed 288 weeks, 1 patient (7.7%) 198 weeks, 4 patients (30.8%) 188 weeks, 3 patients (23.1%) 164 weeks, and 4 patients 159, 156, 92, and 52 weeks, respectively. All patients were compliant with idursulfase treatment and the compliance with weekly infusions was 100%. All infusions were administered in hospital settings.

### Clinical studies (measures of skeletal growth)

**Figure 1:**
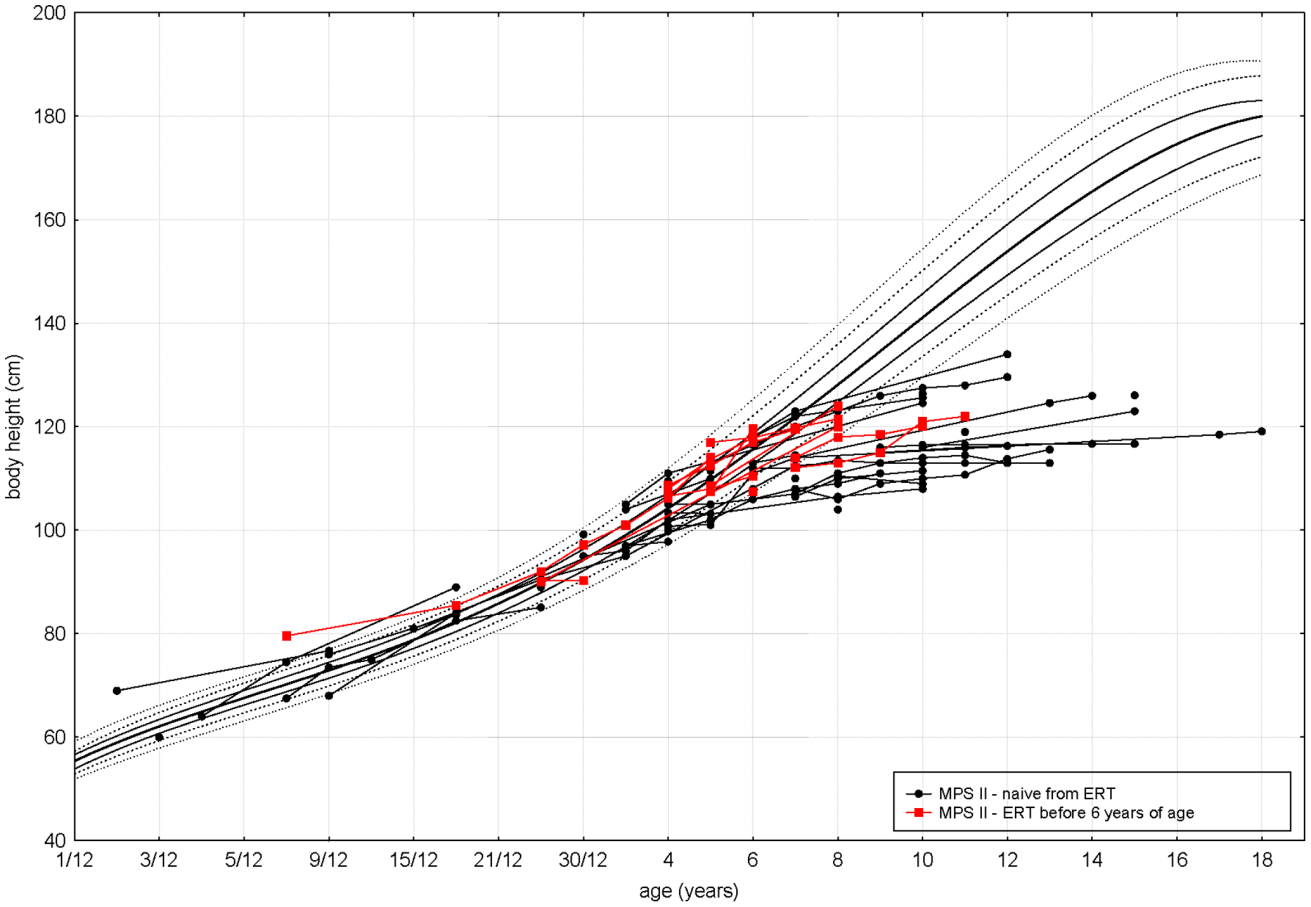
Growth curves for patients with MPS II who started ERT before 6 years of age (red marks) and patients naïve to ERT (black marks) on references growth charts for healthy population.

**Figure 2:**
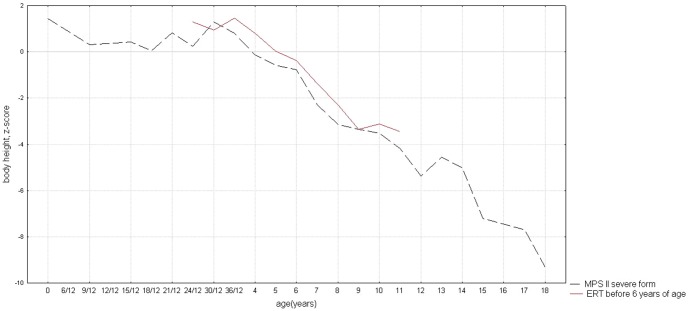
Comparison of growth patterns of patients with mucopolysaccharidosis type II who started enzyme replacement therapy before 6 years of age and patients who were naïve to ERT.

Figure 1 presents individual growth curves for patients from group 1 and 2 on the reference growth charts. Individual data for this study was standardized in order to show the actual degree and direction of deviations compared with the Polish reference charts. Figure 2 presents the mean standardized values for the body height in the calendar age groups. The results show the existence of characteristic downward trend in degree and direction of deviations in MPS II boys who started enzyme replacement therapy before 6 years of age. This course of average growth curve for MPS II ERT group was similar to growth pattern in MPS naïve group. Although the average z-score value of body height in subsequent years in MPS II ERT group was a bit greater than in MPS II naïve group this difference was not statistically significant.

## Discussion

There is a scarcity of literature reporting detailed anthropometric data of both untreated children with MPS disorders as well as children receiving ERT [8]–[10], [13] with only two studies dealing exclusively with impact of ERT on growth (Table 2) [9], [10].

**Table 2:** Comparison of studies addressing effect of ERT on growth in patients with mucopolysaccharidosis type II.

	Schulze et al, 2011	Jones et al, 2013	Current study
Study group	18	133	13
	Germany & UK	different countries around the world	Poland
Age at ERT	2 groups	variable	< 6 y
introduction	< 10 y, > 10 y		
Severity of the disease	attenuated	?	neurological (88%) attenuated (7.7%)
Method	no detailed description	no detailed description	one anthropology specialist, precise measurements
z-score	calculated in comparison to the CDC growth charts	calculated in comparison to the CDC growth charts	calculated in comparison to the regional reference charts
Age classes	yes	no	yes
Data analysis			
Conclusions	ERT seemed to have a positive influence on growth in patients with MPS II with most benefit in patients beginning ERT before the age of 10 years.	Idursulfase treatment improves growth rate in patients with Hunter syndrome.	Idursulfase did not appear to alter the growth patterns.

Schulze et al compared annual growth rates before and during ERT in 18 patients. Group 1 included nine patients who started ERT before 10 years of age; group 2 contained nine patients aged more than 10 years at the start of ERT [9]. All patients had received weekly or biweekly ERT or placebo for 1 year, followed by ERT for more than 3 years. For patients in group 1, the mean (±SD) height increase was 14.6±5.5 cm during 3 years of ERT. Patients in group 2 had a mean height increase of 8.1±1.7 cm after 3 years of ERT compared with an increase of 1 cm in the year before ERT [9]. The authors concluded that ERT seemed to have a positive influence on growth in patients with MPS II with most benefit in patients beginning ERT before the age of 10 years.

In a recent study, Jones et al, performed an analysis to investigate the effects of ERT on growth in patients enrolled in HOS – the Hunter Outcome Survey. This study showed that the slope after treatment (slope = –0.005) was significantly improved compared with before treatment (slope = –0.043) (difference = 0.038, p = 0.004) [10]. Analysis of covariates (age at treatment start, cognitive involvement, presence of puberty at the start of ERT, mutation type, functional classification), showed a significant influence on growth of mutation type (height deficit in terms of z-scores most pronounced in patients with deletions/large rearrangements/nonsense mutations, p<0.0001) and age (most pronounced in 12–15-year group, p<0.0001) [10]. The authors concluded that idursulfase treatment improves growth rate in patients with Hunter syndrome.

The results of the above-mentioned studies do not seem consistent with our results.

In our study of 13 patients with MPS II all measurements were performed by the same anthropology specialist using appropriate equipment and based on appropriate general population standards. The majority of our patients (85%) received ERT for at least 156 weeks. One of the key observations from comparing treated and naïve patients is that there was no statistically significant difference in the mean increase in body height between these two groups.

Although, some authors suggested improvement in growth rates in patients treated with ERT, our data does not support this notion. The reason for this difference may be a less detailed assessment and shorter observation time in other studies (Table 2). In large international studies, height is measured in different hospitals, by different people, which may have an impact on the quality of measurements. What’s more, study groups are very heterogenous, for example in a study by Jones et al study group consisted of 892 males form 116 different countries around the world. These patients have not been divided into different groups depending on their IQ. Another often occurring problem is z-score, which is calculated in comparison to the CDC growth charts. The most recent publications, which focus on the problem of using international standards, show that global growth charts are inadequate in comparison to the regional charts [14]–[18]. Z-score should be calculated based on reference standards valid for the country of origin of the patient.

Body height, defined as a measurement of total body length in an upright position, describes the complicated structure of ontogeny. Process of growth is non- linear with both periodic and chaotic elements, which are specific for growth pattern in human species. Velocity and rate of growth change during ontogeny and growth rate differs in particular age classes. Generally, growth can be characterized as a period of accelerated growth divided by periods of slow growth. Individuals grow according to genetically determined pattern. The growth pattern contains the following stages: infancy with rapid postnatal spurt immediately after birth; childhood followed by a small mid-growth spurt at the age of 6–7 years; juvenile and adolescence with its marked pubertal growth spurt. Patients with MPS II, despite the disease, show the same general growth pattern, characteristic for the human species. In connection with the above-mentioned facts, it is a misapprehension to analyze growth of chronically ill patients assuming that: the rate of growth is constant (study by Schulze-Frenking) or the slope of z-score values is constant in subsequent years (HOS study) [9], [10]. It is also inappropriate to analyze the process of growth for all individuals with different age in one age group like in HOS study (8–15 years). Between 12 to 15 years there is a marked acceleration of growth in human growth pattern and ignoring this fact makes the above-mentioned studies unconvincing. Growth process in MPS II patients is undoubtedly different from the growth pattern in healthy population and therefore the most appropriate method of analyze the effects of treatment is to compare the growth of ERT treated patients with patients naïve to ERT.

It is important to remember that ERT is a treatment with partial effects in specific body compartments [19]. Although it can be a life-lengthening therapeutic measure, it does not seem to alter the natural history of the skeletal disorder in MPS II and children will still endure the skeletal changes of the disease.

## Conclusion

No apparent difference was observed between the growth rates in treated patients and the untreated disease. New approaches for the management and treatment of MPS disorders are necessary to influence skeletal abnormalities.
